# Analysis of the Spatial Distribution and Common Mode Error Correlation in a Small-Scale GNSS Network

**DOI:** 10.3390/s24175731

**Published:** 2024-09-03

**Authors:** Aiguo Li, Yifan Wang, Min Guo

**Affiliations:** School of Surveying and Land Information Engineering, Henan Polytechnic University, Jiaozuo 454000, China; lag18@126.com (A.L.); guominsj@126.com (M.G.)

**Keywords:** GPS coordinate time series, common mode error separation, independent component analysis, correlation coefficient

## Abstract

When analyzing GPS time series, common mode errors (CME) often obscure the actual crustal movement signals, leading to deviations in the velocity estimates of station coordinates. Therefore, mitigating the impact of CME on station positioning accuracy is crucial to ensuring the precision and reliability of GNSS time series. The current approach to separating CME mainly uses signal filtering methods to decompose the residuals of the observation network into multiple signals, from which the signals corresponding to CME are identified and separated. However, this method overlooks the spatial correlation of the stations. In this paper, we improved the Independent Component Analysis (ICA) method by introducing correlation coefficients as weighting factors, allowing for more accurate emphasis or attenuation of the contributions of the GNSS network’s spatial distribution during the ICA process. The results show that the improved Weighted Independent Component Analysis (WICA) method can reduce the root mean square (RMS) of the coordinate time series by an average of 27.96%, 15.23%, and 28.33% in the E, N, and U components, respectively. Compared to the ICA method, considering the spatial distribution correlation of stations, the improved WICA method shows enhancements of 12.53%, 3.70%, and 8.97% in the E, N, and U directions, respectively. This demonstrates the effectiveness of the WICA method in separating CMEs and provides a new algorithmic approach for CME separation methods.

## 1. Introduction

High-precision GNSS coordinate time series have gradually become an important tool in geoscientific research, such as studying plate motion and monitoring crustal deformation [1,2,3,4]. Continuous GNSS coordinate sequences include not only tectonic signals but also other non-tectonic signals and unmodeled error sources, all of which can interfere with the estimation and analysis of station coordinates and velocities [5,6,7]. There is a major spatiotemporal error source among GPS stations that significantly affects the accuracy and reliability of station positioning. Therefore, in GNSS coordinate time series analysis, mitigating the impact of common mode errors (CME) on positioning accuracy and identifying effective tectonic signals from the time series are key research targets to ensure the precision and reliability of GNSS time series [8,9,10,11]. However, there is no consensus on the physical origin of CME. It is generally believed that the CME in GPS coordinate series primarily originates from unmodeled systematic errors, environmental impacts around the stations, and is mainly manifested as correlations between stations [12,13,14]. Research on common mode errors has mainly focused on regional GPS observation networks [7,15,16]. Additionally, some scholars believe that as the distance increases, the impact of CME between stations gradually decreases [17,18,19]. Beyond approximately 2000 km, the correlation diminishes, and the errors introduced by the reference frame replace CME as the primary source of error [20]. Based on this, to separate CME in large-scale GPS networks, the SOPAC center proposed a subnet filtering method, which divides the GPS observation network into several small regions, and then separates the CME in each small region [21]. Therefore, improving the accuracy of CME separation in small regions is of great significance for the study of CME separation.

To eliminate or reduce the impact of CME in coordinate series, many scholars have conducted a series of studies on CME separation. Wdowinski was the first to observe CME in his research on earthquakes in Southern California and proposed the regional stacking filter method to separate CME. This method focuses on the coordinate time series of single stations and single components, ignoring spatial correlations caused by various factors [22]. The Correlation Coefficient Weighted Stacking Filter is an improvement over the Regional Stacking Filter, using correlation coefficients as weight factors to account for spatial distribution differences among stations [19,23,24]. Nevertheless, traditional methods fail to completely separate multiple signals with close frequencies in the time series [14,25,26]. Dong et al. proposed Principal Component Analysis (PCA) and Karhunen-Loeve (KLE) to address the shortcomings of traditional methods [13]. Additionally, Tian and Shen experimentally verified that the positions and distances of adjacent stations affect the accuracy of station time series and designed a grid search algorithm for optimization [12]. Zhou proposed using Multichannel Singular Spectrum Analysis to extract CME [27]. Ming compared the effectiveness of ICA and PCA methods in separating CME and validated the feasibility of the ICA algorithm through practical cases [28,29]. ICA is a blind source separation method that processes time series by introducing higher-order statistics to separate statistically independent non-Gaussian signals [30,31,32]. Bai et al. experimentally analyzed that PCA is not suitable for extracting CME in large spatial scale GPS networks, while ICA provides better filtering results [33].

ICA is a non-parametric method used to separate and extract CME from GNSS time series data. Typically, ICA analyzes individual components without accounting for the spatial correlations between stations. In this study, GPS coordinate time series data provided by the Nevada Geodetic Laboratory was used to separate CME. We employed a variant of the ICA method known as Weighted Independent Component Analysis (WICA). This modification improves the CME extraction process by incorporating correlation coefficients between stations as weight factors. By doing so, WICA enhances or diminishes the contribution of the GNSS network’s spatial distribution during the ICA process, thereby considering the correlations between stations while separating the CME.

The remainder of this paper is structured as follows: Section 2 presents the GNSS data sources as well as preprocessing and processing methods. Section 3 discusses the relationship between the spatial distribution of GNSS stations and common mode errors within a small region, as well as the effect of weighting the data in ICA to emphasize or diminish the contribution of the GNSS network’s spatial distribution in CME separation. Section 4 discusses the experimental results, and Section 5 provides the key conclusions.

## 2. Materials and Methods

### 2.1. GNSS Data Source

**Figure 1:**
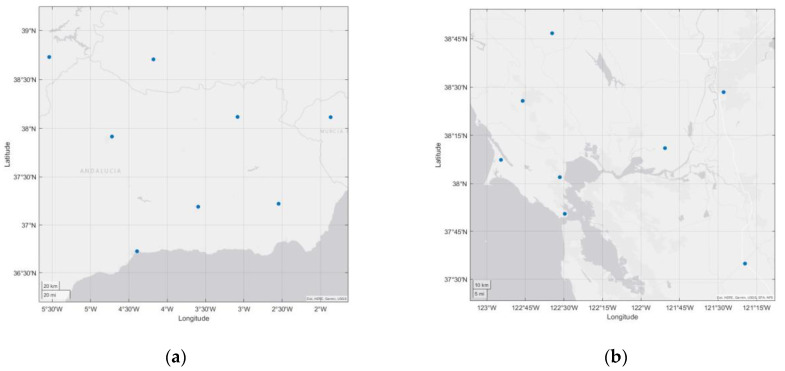
Experiment Area 1 is located in southern Spain, while Experiment Area 2 is situated on the west coast of the United States. Both areas are coastal. (**a**) Experiment Area 1. (**b**) Experiment Area 2. The blue dots represent the spatial distribution of stations in the test area.

To analyze the relationship between CME and the spatial correlation of GNSS stations in a small area, while simplifying the process of data handling and analysis and excluding other incidental factors, two experimental regions were selected from the GNSS data provided by the Nevada Geodetic Laboratory. Each region included continuous GPS time series data from eight GPS stations covering the period from 2014 to 2022. This setup allowed for the comparative validation of the spatial distribution of GNSS stations and their correlation with CME. The latitudes of the experimental areas range from 37 °N to 39 °N, and they share similar climatic and geological conditions. The distribution of GPS stations is shown in Figure 1, and the station coordinates are the average values of the coordinates of each station, as shown in Table 1 and Table 2.

**Figure 2:**
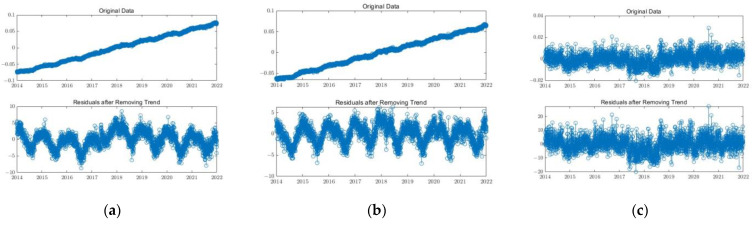
The original time series and the detrended time series of station GRA1 in three directions. (**a**) E component. (**b**) N component. (**c**) U component.

In this study, the triple median error method was first applied to remove outliers from the time series of each station [34]. Then, cubic polynomials were used for interpolation and to fill in missing data [35]. Finally, the mean, velocity, and step terms were removed from the original time series [36,37,38] to obtain the residual time series for each GPS station. The uncertainties of all GPS stations with CME were retained and filtered. Figure 2 shows the original time series and the detrended time series of the GRA1 station.

### 2.2. Common Mode Error Analysis Methods

#### 2.2.1. Regional Stacking Filter Method

The regional stacking filter method is a commonly used approach for obtaining CME. This method assumes that the CME is identical for each station and uses the error from daily solutions as a weighting factor to separate the CME [22]. Assuming that the CME is uniformly distributed within a certain space, it can be expressed as:

(1) $$\varepsilon_{i}=\frac{\sum_{S=1}^{S_{i}}\frac{v_{i,j}}{\sigma_{i,j}^{2}}}{\sum_{S=1}^{S_{i}}\frac{1}{\sigma_{i,j}^{2}}}$$

In the formula, $v_{ij}$ represents the residual between two stations, and $\sigma_{i,j}$ denotes the standard error of the j-th station on the i-th day.

#### 2.2.2. Correlation Coefficient Weighted Stacking Filter

The Correlation Coefficient Weighted Stacking Filter is an improved method based on the Region-Based Stacking Filter. It introduces the correlation coefficient as a weighting factor to account for the spatial distribution differences among stations.

This method enhances the traditional Region-Based Stacking Filter by incorporating the spatial correlation between stations. In the traditional method, it is assumed that CME is the same for all stations, and the error of each daily solution is used as a weighting factor to separate CME. However, this assumption does not consider the spatial distribution of the stations. The Correlation Coefficient Weighted Stacking Filter improves upon this by incorporating the spatial correlation between stations [19,23,24].

(2) $$r_{xy}=\frac{\sum_{k=1}^{N}\left(x_{k}-\bar{x}\right)\left(y_{k}-\bar{y}\right)}{\sqrt{\sum_{k=1}^{N}{\left(x_{k}-\bar{x}\right)}^{2}\sum_{k=1}^{N}{\left(y_{k}-\bar{y}\right)}^{2}}}$$

In the formula, $r_{ij}$ represents the correlation coefficient between two stations, N represents the number of common epochs; $x_{k},\;y_{k}$ represent the residual coordinate time series of two GPS reference stations; ${\bar{x}}_{k},{\bar{y}}_{k}$ represent the mean values of the residual coordinate time series for the two GPS reference stations.

(3) $$\varepsilon_{i}=\sum_{S=1}^{S_{i}}\frac{v_{i,j}}{\sigma_{i,j}^{2}}\times \frac{r_{ij}}{\sum_{S=1}^{S_{i}}\frac{1}{\sigma_{i,j}^{2}}\left|r_{ij}\right|}$$

In the formula, $r_{ij}$ represents the correlation coefficient between two stations, $v_{ij}$ represents the residual between two stations, and $\sigma_{i,j}$ denotes the standard error of the j-th station on the i-th day.

#### 2.2.3. Correlation Coefficient Weighted Independent Component Analysis

ICA (Independent Component Analysis) is a blind signal separation method that can be used to extract high-order information from coordinate time series. Given that CME exhibits spatial correlation, this paper takes into account the relationship between the spatial distribution of small-scale GNSS network stations and common mode error. We improve the independent component analysis method by introducing the correlation coefficient as a weighting factor, which emphasizes or de-emphasizes the contribution of the GNSS network’s spatial distribution during the ICA process [30,31,32].

For an area with n observation stations conducting continuous observations for m days (m>n), the detrended and demeaned m×n residual time series can be represented as X. The mathematical model of ICA can be expressed as:

(4) $$X_{n\times m}=A_{n\times t}S_{t\times m}$$

In this equation: X represents the observation matrix, A represents the mixing matrix, S represents the source signal matrix.

Building on the ICA method, we locate the station at the spatial geometric center, calculate the correlation coefficients between the geometric center station and other stations, and define this correlation coefficient matrix as P. Assuming the geometric center station is $i_{c}$, for each station j, we calculate the correlation coefficient $r_{ij}$ between the geometric center station ${\;i}_{c}\;$ and station j. The elements $p_{ij}$ of matrix P are defined as the correlation coefficients $r_{i_{c}j}$ between the geometric center station $i_{c}$ and station j. The calculation method for the correlation coefficient in P is consistent with Equation (2). The correlation coefficient matrix P is defined as:

(5) $$P=diag[r_{i_{c}j_{1}}\;\;r_{i_{c}j_{2}\;}\;\;r_{i_{c}j_{3}}\cdots \;\;r_{i_{c}j_{n}}]$$

where n is the total number of stations. The original data is then weighted using P.

(6) $$C={P_{n\times n}X}_{n\times m}$$

In the centered and whitened matrix, the covariance matrix can be defined as B.

(7) $$B=\frac{1}{m}C^{T}C$$

For the covariance matrix B, performing orthogonal decomposition yields:

(8) $$B=V\Lambda V^{T}$$

In the expression, $V^{T}$ represents the matrix of eigenvectors. Λ is a diagonal matrix with r non-zero eigenvalues (n≥r).

Then, whitening transformation is performed to further separate the mixed signals independently. The whitening process involves dimensionality reduction and transformation combinations to separate mixed signals and reduce the data’s dimensionality, especially when the data contains non-Gaussian mixed signals. After the observational data is centralized and whitened, matrix z is obtained. Afterward, the original signals are recovered using the demixing matrix W, and the process is iterated until convergence. The formula is as follows:

(9) $$w_{i}=E\left\{zg\left(w_{i}^{T}z\right)\right\}-E\left\{g^{\prime }\left(w_{i}^{T}z\right)\right\}w$$

In the formula, $g(s)=\frac{1}{a_{1}}\log(\cos a_{1}y)$,${\;g}^{\prime }\left(s\right)=\tan\left(a_{1}y\right)$, where $a_{1}$ is a constant with $1\leq a_{1}\leq 2$ and is typically set to 1.

Through the previous step, we obtain the approximated matrix Y. From this matrix, we select the independent components that exhibit similar patterns as the common mode error.

(10) $$\varepsilon =\sum_{k=1}^{l}a_{k}s_{k}$$

In the equation, ε represents the common mode error, while $a_{k},\;s_{k}$ denote the spatial response and temporal response of the k-th principal component, respectively.

**Figure 3:**
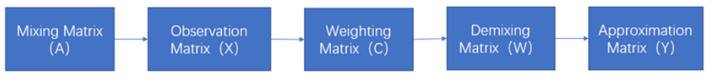
The process of the improved WICA method.

**Figure 4:**
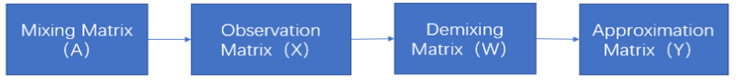
The processing procedure of the ICA method.

The process of the improved WICA method is shown in Figure 3:

Figure 4 illustrates the process of the Independent Component Analysis (ICA) method. The primary objective of ICA is to recover the original source signals, denoted as S in Equation (4), from the observed mixed signals. This is achieved by estimating the unmixing matrix W, which is the inverse of the mixing matrix A. The estimation of W is performed through an iterative process, where the matrix is updated progressively until it converges to a stable solution. Once convergence is achieved, the resulting unmixing matrix W can be used to separate the mixed signals into their independent components, yielding the approximated source signals represented by the matrix Y.

The key difference between the improved method and the traditional ICA method lies in the consideration of spatial distribution differences among stations. The improved method employs correlation coefficients to weight the data, specifically by assigning greater weight to stations with higher correlation, and then using filtering techniques to separate CME, thereby enhancing the estimation of CME. Specifically, before calculating the unmixing matrix W, the observation matrix X is first weighted using the correlation coefficient matrix P, which is calculated according to Equation (2). The weighted observation matrix is then used in the subsequent unmixing process to finally obtain the approximation matrix.

## 3. Results

### 3.1. Analysis of Common Mode Error Correlation among Stations

**Figure 5:**
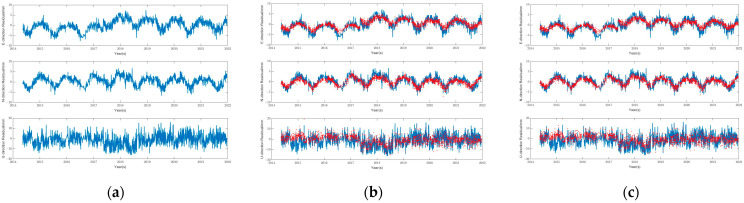
Filtering results before and after using regional stacking filter and correlation coefficient weighted stacking filter methods. (**a**) Before filtering. (**b**) After filtering with regional stacking filter. (**c**) After filtering with correlation coefficient weighted stacking filter.

To analyze and verify the relationship between the generation of CME and the spatial distribution of stations, two sets of experiments were designed for comparison. The correlation coefficients of residual coordinate time series between the central station and other stations in the two test areas were calculated before and after filtering. Figure 5 shows the effects before filtering and after applying the Regional Stacking Filter Method and the Correlation Coefficient Weighted Stacking Filter Method, with the red sections representing the results after filtering using both methods. By comparing the images before and after spatiotemporal filtering, it can be observed that the periodicity of each coordinate time series has been weakened to a certain extent after filtering. This indicates that, after separating the CME, the coordinate time series can effectively reduce the errors in estimating linear and periodic terms, thereby improving the overall accuracy of the coordinate time series. This also suggests that CME is a contributing factor to the periodic fluctuations observed in GNSS coordinate time series. Additionally, it can be observed from the figures that there are significant variation patterns and periodicity in the N and E directions, while similar phenomena are not evident in the U direction. This is because the signal paths of the GPS station coordinate residual time series in the horizontal directions are more easily affected by environmental and surface movements, leading to more pronounced periodic variations in the residual time series in the N and E directions. In contrast, the vertical direction is generally less influenced by such factors.

Table 3 shows the mean and change rate of the correlation coefficients between the geometric center station and other stations in the two experimental areas before and after extracting the CME using the regional stacking filter method. The geometric center station for Experiment Area 1 is GRA1 (37.19, −3.59), and for Experiment Area 2 is SVIN (38.03, −122.52). The results indicate a significant reduction in the correlation between stations after separating the CME. Before filtering, the correlation coefficients in all three directions for Experiment Area 1 are larger than those for Experiment Area 2. After filtering, the average reduction in the correlation coefficients in the N direction is the greatest for both experimental areas.

Before and after filtering, the correlation coefficients in Experiment Area 1 have decreased. The reduction is more significant in the E and U directions compared to the N direction. Specifically, in the E direction, the mean correlation coefficient decreased from 0.4963 to 0.3356. In the N direction, the coefficient still decreased slightly, with the maximum value dropping from 0.3722 to 0.3717. In the U direction, the coefficient decreased from 0.4802 to 0.2882. In Experiment Area 2, the correlation coefficients in all three directions showed significant reduction after filtering. In the E direction, the mean correlation coefficient decreased from 0.7129 to 0.3539. In the N direction, it dropped from 0.5661 to 0.3770. In the U direction, the coefficient decreased from 0.5483 to 0.2870. As before filtering, the correlation coefficients between each station and the geometric center station vary in the three directions.

The Correlation Coefficient Weighted Overlay Filtering method is an improvement on the Regional Overlay Filtering method, using correlation coefficients as weighting factors to account for spatial distribution differences among stations. Table 3 shows the changes in correlation coefficients between the geometric center station and other stations after applying the Correlation Coefficient Weighted Overlay Filtering. After this method, the correlation coefficients in all three components have been reduced to some extent.

In Experiment Area 1, the mean correlation coefficients before and after filtering are as follows: In the E direction, the mean decreased from 0.4963 to 0.3042; in the N direction, the coefficient dropped from 0.3722 to 0.3624; and in the U direction, it decreased from 0.4802 to 0.2829. In Experiment Area 2, the mean correlation coefficients in all three directions after filtering are: In the E direction, the mean decreased from 0.7129 to 0.3257; in the N direction, it dropped from 0.5661 to 0.3712; and in the U direction, it reduced from 0.5483 to 0.2537. Compared to the Regional Overlay Filtering method, considering the spatial distribution differences among the stations has further reduced the values of the correlation coefficients.

Based on the spatial distribution correlation coefficients of the stations, this study also uses the root mean square (RMS) of the residual coordinate time series to assess the quality and improvement rate of different filtering methods before and after filtering. A reduction in the RMS of the GNSS residual coordinate time series after CME separation indicates a decrease in the dispersion of the GNSS residual coordinate time series, reflecting better optimization.

Table 4 shows the changes in RMS values of the stations before and after filtering using the Regional Stacking Filter Method and the Correlation Coefficient Weighted Stacking Filter Method. It can be seen that the RMS values of the stations in both experimental areas decreased after separating the common mode errors using these two filtering methods, indicating a reduction in data dispersion. Notably, the RMS values after filtering using the Correlation Coefficient Weighted Stacking Filter Method are smaller than those after filtering using the Regional Stacking Filter Method, suggesting that common mode errors are related to the spatial distribution of stations. The differences in spatial distribution can effectively separate out common mode errors. Furthermore, as seen in Table 3, the correlation coefficients before and after filtering show that the correlation coefficients in the three directions before filtering in Experimental Area 2 are 0.71, 0.56, and 0.54, respectively, while in Experimental Area 1, they are 0.49, 0.37, and 0.48. The correlation coefficients between stations in Experimental Area 2 are higher than those in Experimental Area 1. Before filtering, the RMS values in the N and U directions of Experimental Area 2 are greater than those in Experimental Area 1, with similar RMS values in the N direction. After removing the common mode errors, the comparison of Table 3 and Table 4 shows that the correlation between the reference stations weakens, and the correlation coefficients decrease accordingly.

### 3.2. Improved Weighted Independent Component Analysis Method

**Figure 6:**
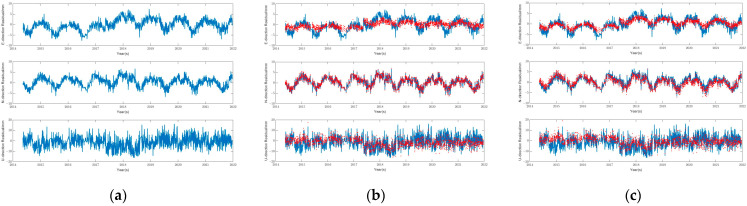
Filtering results before and after using ICA and WICA methods. (**a**) Before filtering. (**b**) After filtering with ICA. (**c**) After filtering with WICA.

Since the residual time series contains other signals in addition to the common mode error and does not follow a Gaussian distribution, compared to the regional stacking method and the correlation coefficient weighted stacking method, the ICA method is theoretically more rigorous for spatiotemporal filtering of GPS time series. The ICA method separates the common mode error through blind source signal separation, making full use of higher-order statistics. By performing independent signal decomposition, it selects the quantities with consistent spatial response (SR) from the independent components (IC) as the common mode error. Figure 6 shows the effects before and after filtering.

As shown in the experiments in Section 3.1, the common mode error is related to the spatial distribution of the stations. Considering the spatial distribution differences of the stations can effectively separate the common mode error. This paper, based on the ICA method, takes into account the spatial distribution differences of the stations and uses correlation coefficients to weight the data. Table 5, Table 6 and Table 7 present the RMS values before and after filtering in Experiment Area 1 using both the ICA method and the improved WICA method.

Table 5, Table 6 and Table 7 shows the average RMS and average RMS reduction for all stations’ residual coordinate time series in Experiment Area 1 before and after filtering. After filtering with ICA, the average reductions in RMS for the E, N, and U components were 15.43%, 11.53%, and 19.36%, respectively, with the U component showing better results than the N and E components. When filtering using the WICA method, the average reductions in standard deviation for the E, N, and U components were 27.96%, 15.23%, and 28.33%, respectively. Compared to the ICA method, after considering the spatial distribution correlation of the stations, the improved WICA method showed improvements of 12.53%, 3.70%, and 8.97% in the E, N, and U directions, respectively. This demonstrates that the WICA method is effective in separating common mode errors, providing a new algorithmic approach for CME separation.

## 4. Discussion

CME are a type of error in GNSS time series that can obscure the actual crustal movement signals when analyzing GPS time series, leading to deviations in research results. It is widely believed that CME may be caused by factors such as atmospheric hydrology, crustal deformation, orbital models, ionospheric models, and the software and algorithms used for coordinate data processing. CME typically does not reflect long-term tectonic movement trends but manifests as highly correlated errors over short periods. If the variation patterns between stations are very consistent, especially over short periods with significant changes, it indicates a high likelihood of CME effects in the region. The current main approach to separating common mode errors from GPS time series is to use signal filtering methods to process the time series data. Through filtering methods, low-frequency and high-frequency signals in GNSS time series are extracted, with low-frequency signals usually related to tectonic plate movements and high-frequency signals potentially related to CME. If multiple station time series show high correlation, it may indicate the presence of common mode errors or shared tectonic movements. By assigning greater weight to highly correlated stations and then using filtering methods to extract low-frequency and high-frequency signals from GNSS time series, the estimation of common mode errors can be improved.

Therefore, this paper has improved the ICA method by introducing correlation coefficients as weighting factors to process the data, considering the contribution of station spatial distribution and effectively enhancing the separation of common mode errors. However, despite the significant improvement with the modified WICA method, this study has some limitations. The research area used in this experiment is relatively small, and further validation is needed for large-scale GNSS networks.

## 5. Conclusions

This study selected stations from two different regions to compare the correlation coefficients and RMS values before and after applying the correlation coefficient-weighted stacking filter method. In Test Area 1, the correlation coefficients in the E, N, and U directions before filtering were 0.49, 0.37, and 0.48, respectively, while in Test Area 2, they were 0.71, 0.56, and 0.54, respectively. The average RMS value in all three directions before filtering was 3.43 mm for Test Area 1 and 3.83 mm for Test Area 2, indicating that Test Area 2 had a generally higher RMS value. After applying the correlation coefficient-weighted stacking filter method, the correlation coefficients in the E, N, and U directions for Test Area 1 were reduced to 0.30, 0.36, and 0.28, respectively, and for Test Area 2, they were reduced to 0.32, 0.37, and 0.25. This demonstrates that if the spatial correlation between reference stations is strong, their common mode errors are also significant, and after removing these errors, the correlation coefficients between the reference stations correspondingly decrease.

By comparing the separation of common mode errors using the regional stacking filter method and the correlation coefficient-weighted stacking filter method, taking Test Area 2 as an example, it can be seen that after considering the spatial distribution of the stations, the change rates of the correlation coefficients in the three directions increased from 50.35%, 33.40%, and 47.65% to 54.31%, 34.42%, and 53.72%, respectively. The RMS values using the regional stacking filter method in the E, N, and U directions decreased from 1.70 mm, 1.54 mm, and 3.90 mm to 1.67 mm, 1.53 mm, and 3.73 mm after applying the correlation coefficient-weighted stacking filter method. This indicates that after accounting for the spatial distribution of stations, both the correlation coefficients and the RMS values are reduced. It demonstrates that by assigning greater weight to stations with high spatial correlation and considering the differences in their spatial distribution, the filtering method can effectively separate common mode errors.

Based on the ICA method, by considering the spatial distribution differences of stations and applying correlation coefficients as weights, the improved WICA algorithm achieved spatial-temporal filtering. The root mean square (RMS) decreased by an average of 27.96%, 15.23%, and 28.33% in the E, N, and U components, respectively. This effectively reduces the periodic variations caused by common mode errors in the time series of each station and significantly lowers the correlation coefficients after filtering. Compared to the ICA method, the improved WICA method shows improvements of 12.53%, 3.70%, and 8.97% in the E, N, and U directions, respectively, when considering the spatial distribution correlation of stations.

## Figures and Tables

**Table 1:** The station coordinates for Experiment Area 1.

Station Name	Latitude	Longitude	Height
ALMO	38.706	−4.18	743.351
CAAL	37.221	−2.548	2210.684
CATU	38.73	−5.539	538.65
COBA	37.916	−4.721	202.074
CRVC	38.115	−1.869	738.087
GRA1	37.19	−3.596	823.247
MALA	36.726	−4.394	119.83
VICA	38.118	−3.083	852.446

**Table 2:** The station coordinates for Experiment Area 2.

Station Name	Latitude	Longitude	Height
MHDL	37.842	−122.494	65.889
P193	38.123	−122.908	66.598
P197	38.429	−122.767	0.033
P206	38.778	−122.576	283.607
P255	37.582	−121.325	73.512
P266	38.184	−121.844	22.449
P268	38.474	−121.464	−23.906
SVIN	38.033	−122.526	−27.561

**Table 3:** Correlation coefficients and their change rates before and after filtering using two methods.

Experimental Area	Experimental Area 1			Experimental Area 2		
Direction	E	N	U	E	N	U
Mean correlation coefficient before filtering	0.49	0.37	0.48	0.71	0.56	0.54
Mean correlation coefficient after filtering using the regional stacking filter.	0.33	0.37	0.28	0.35	0.37	0.28
Change rate of the mean correlation coefficient after filtering using the regional stacking filter/%.	32.37	0.13	39.98	50.35	33.40	47.65
Mean correlation coefficient after filtering using the correlation coefficient weighted stacking method.	0.30	0.36	0.28	0.32	0.37	0.25
Change rate of the mean correlation coefficient using the correlation coefficient weighted stacking method/%.	38.70	2.63	41.08	54.31	34.42	53.72

**Table 4:** RMS values before and after filtering using Regional Stacking Method and Correlation Coefficient Weighted Stacking Method.

Experimental Area	Experimental Area 1			Experimental Area 2		
Direction	E	N	U	E	N	U
RMS before filtering (mm)	1.92	2.52	5.85	2.99	2.43	6.08
RMS using Regional Stacking Filter (mm)	1.43	2.19	4.24	1.70	1.54	3.90
RMS using Correlation Coefficient Weighted Stacking Filter (mm)	1.40	2.18	4.20	1.67	1.53	3.73

**Table 5:** RMS values in the E direction before and after filtering using ICA and WICA Methods.

Method	Station								Filtering Rate/%
	ALMO	CAAL	CATU	COBA	CRVC	GRA1	MALA	VICA	
RMS before filtering (mm)	1.7949	1.9421	1.5079	1.5185	1.9404	2.5487	2.6103	1.515	
RMS using ICA (mm)	1.034	1.74	1.4853	1.4128	1.6331	2.0691	2.5919	1.1079	15.43
RMS using WICA (mm)	0.9254	1.6286	0.8645	1.1902	1.7138	1.6344	2.4235	0.90627	27.96

**Table 6:** RMS values in the N direction before and after filtering using ICA and WICA Methods.

Method	Station								Filtering Rate/%
	ALMO	CAAL	CATU	COBA	CRVC	GRA1	MALA	VICA	
RMS before filtering (mm)	3.4761	3.4241	1.6881	2.0365	2.3258	2.2333	3.1696	1.8095	
RMS using ICA (mm)	2.4014	2.9102	1.6806	2.0083	2.2036	1.5011	2.9996	1.7892	11.53
RMS using WICA (mm)	2.7561	2.736	1.6868	1.8261	2.209	2.1448	1.2765	1.7747	15.23

**Table 7:** RMS values in the U direction before and after filtering using ICA and WICA Methods.

Method	Station								Filtering Rate/%
	ALMO	CAAL	CATU	COBA	CRVC	GRA1	MALA	VICA	
RMS before filtering (mm)	5.2996	6.1776	5.1835	7.0438	5.1321	5.4421	6.7676	5.7815	
RMS using ICA (mm)	4.1702	4.9092	5.066	5.0487	4.0337	4.9144	4.1346	5.0586	19.36
RMS using WICA (mm)	3.8522	3.9428	4.4056	4.0795	4.1465	3.8767	4.1428	4.6632	28.33

## Data Availability

The raw/processed data required to reproduce these findings cannot be shared at this time as the data also form part of an ongoing study.
